# Influence of extracellular matrix composition on tumour cell behaviour in a biomimetic in vitro model for hepatocellular carcinoma

**DOI:** 10.1038/s41598-023-27997-3

**Published:** 2023-01-13

**Authors:** Carlemi Calitz, Jenny Rosenquist, Oliver Degerstedt, Jaafar Khaled, Maria Kopsida, Mårten Fryknäs, Hans Lennernäs, Ayan Samanta, Femke Heindryckx

**Affiliations:** 1 Department of Medical Cell Biology, Uppsala University, Husargatan 3, Box 571, 75431 Uppsala, Sweden; 2 Polymer Chemistry, Department of Chemistry-Ångström Laboratory, Uppsala University, Box 538, 75121 Uppsala, Sweden; 3 Department of Pharmaceutical Biosciences, Uppsala University, Uppsala, Sweden; 4 Department of Medical Sciences, Cancer Pharmacology and Computational Medicine, Uppsala University, Uppsala, Sweden

**Keywords:** Liver cancer, Cancer microenvironment, Cancer models

## Abstract

The tumor micro-environment (TME) of hepatocellular carcinoma (HCC) consists out of cirrhotic liver tissue and is characterized by an extensive deposition of extracellular matrix proteins (ECM). The evolution from a reversible fibrotic state to end-stage of liver disease, namely cirrhosis, is characterized by an increased deposition of ECM, as well as changes in the exact ECM composition, which both contribute to an increased liver stiffness and can alter tumor phenotype. The goal of this study was to assess how changes in matrix composition and stiffness influence tumor behavior. HCC-cell lines were grown in a biomimetic hydrogel model resembling the stiffness and composition of a fibrotic or cirrhotic liver. When HCC-cells were grown in a matrix resembling a cirrhotic liver, they increased proliferation and protein content, compared to those grown in a fibrotic environment. Tumour nodules spontaneously formed outside the gels, which appeared earlier in cirrhotic conditions and were significantly larger compared to those found outside fibrotic gels. These tumor nodules had an increased expression of markers related to epithelial-to-mesenchymal transition (EMT), when comparing cirrhotic to fibrotic gels. HCC-cells grown in cirrhotic gels were also more resistant to doxorubicin compared with those grown in fibrotic gels or in 2D. Therefore, altering ECM composition affects tumor behavior, for instance by increasing pro-metastatic potential, inducing EMT and reducing response to chemotherapy.

## Introduction

Hepatocellular carcinoma (HCC) is a primary liver cancer characterised by poor prognosis and few therapeutic advances^1,2^. It has one of the highest incidences of cancer-related mortality and it is estimated that by 2030 it will result in 1 million deaths annually, specifically due to the increased incidence of obesity and non-alcoholic fatty liver disease^3^. The development of HCC is attributed to fibrosis and cirrhosis, which develops because of chronic liver injury and inflammation. Chronic liver injury and inflammation can be caused by various aetiologies, including viral hepatitis, aflatoxins, excessive alcohol intake, non-alcoholic steatohepatitis, and metabolic syndrome^4^. The underlying fibrosis is an active contributor in the pathogenesis of HCC, as it contributes to a complex interplay between injured hepatocytes, the stromal microenvironment, and alterations in ECM composition, which all contribute to HCC progression and may hamper the response to chemotherapeutics^1,2,5^. Specifically in the context of trans-arterial chemo-embolization with doxorubicin (DOX)-loaded formulations for intermediate-stage HCC-patients, the abundant ECM and substantial accumulation of stromal cells, are considered a binding-site barrier that may reduce effective penetration of drugs towards the active tumour site^2^. However, stromal cells and ECM may also directly influence drug response, by altering gene-expression of cancer cells and pushing them towards a more aggressive tumour phenotype^6,7^.

Hepatic fibrosis is an integral part in the progression of chronic liver disease, ultimately leading to cirrhosis and hepatocellular carcinoma. Cirrhosis is considered the end-stage of liver disease and consists of nodular regeneration and a continuous fibrotic process. This process is characterized by activation of hepatic stellate cells and leads to increased deposition of extracellular matrix (ECM) components, such as collagen^8^. The evolution of liver fibrosis is characterized by major remodelling of the ECM, whereby the initial stages are characterized by a predominant increase of collagen types I and III, while later stages are mediated by the overexpression of proteins involved in network-forming collagen and elastic fibre assembly^9^. Cross-linked elastic fibres are known to be more resistant to matrix metalloproteinases mediated degradation, thus contributing to the irreversibility of cirrhosis^9^. As liver disease progresses, an increased deposition of high molecular weight, crosslinked fibrin(ogen) becomes tightly colocalized with collagen, forming a dense fibrous network^10^. The overall increased deposition of ECM, in addition to its molecular remodelling will result in various biological and physical changes in the microenvironment, including solid stress, increased matrix stiffness and interstitial hydraulic pressure^11^. In a healthy human liver, the stiffness ranges between 0.4 and 0.6 kPa, which during HCC can increase by a tenfold^6,11^. This stiffening of the liver is not only a consequence of fibrosis, but also an active driver of the disease. This is supported by the notion that the elevation of liver stiffness precedes development of fibrosis^12^. In turn, increased stiffness induces a metabolic switch in both tumour and stromal cells altering cellular machinery^6,11^. Alterations in molecular composition of the ECM and the subsequent change in biomechanical properties affects various cellular functions including proliferation, metabolic cellular reprogramming, migration, and invasion as well as chemotherapeutic resistance^6,11,12^.

Effectively mimicking alterations of ECM in different disease states in vitro as well as in vivo has been challenging. However, with the advent of three-dimensional (3D) cell culture models, this has become a more realistic outlook. To that extent, we have previously published a method for creating a 3D biomimetic model with tuneable biomechanical and physical properties that allows us to investigate tumour–stroma interactions^7^. Using this model, we now explore the role of different ECM compositions and their corresponding stiffness on epithelial-to-mesenchymal transitions (EMT), metastatic potential, drug sensitivity and overall proliferation, thereby comparing results to current two-dimensional (2D) models, an in vivo HCC mouse model and available clinical data.

## Results

### Mimicking ECM stiffness seen in fibrosis and cirrhosis

**Figure 1:**
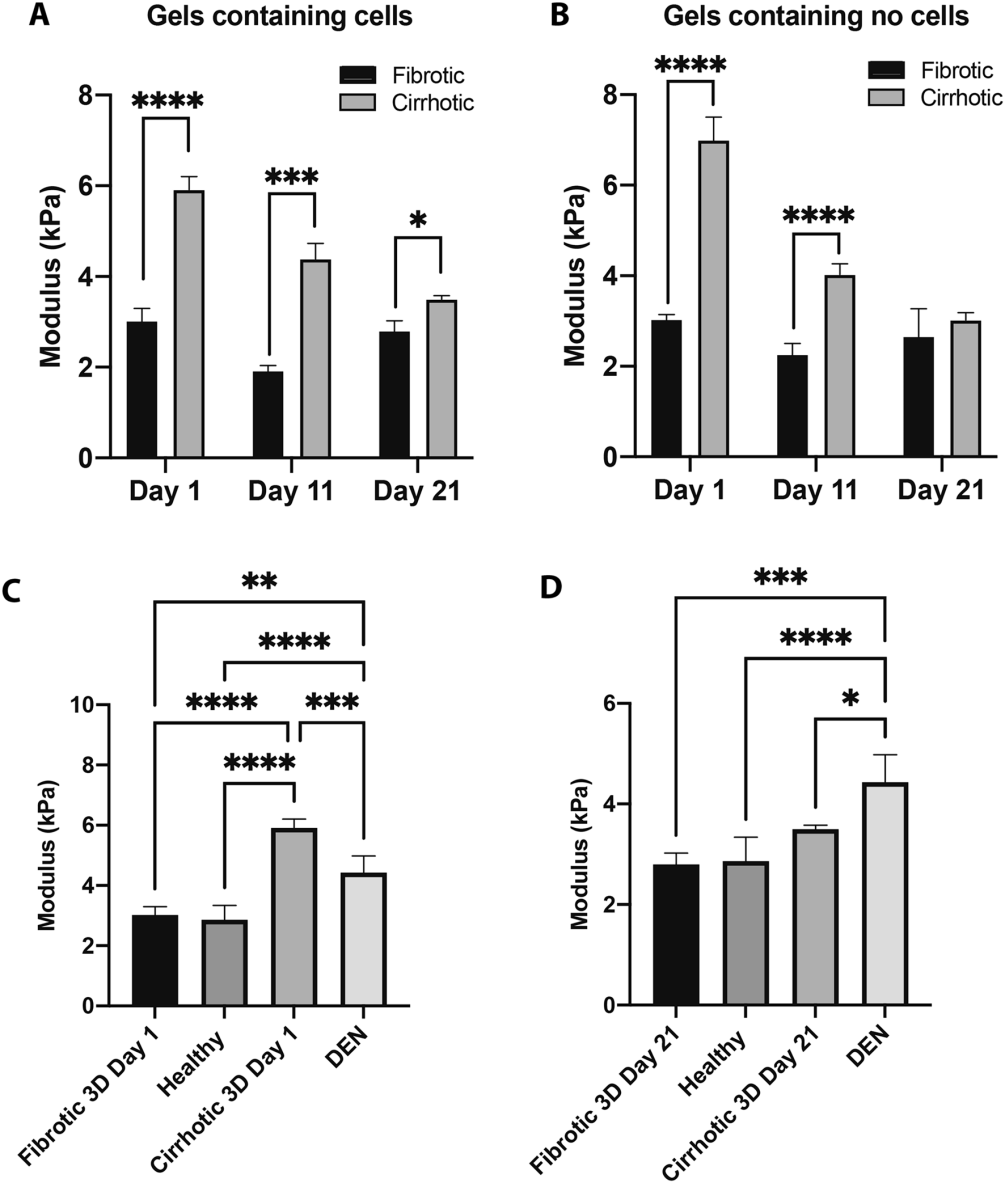
Liver stiffness determined via parallel plate rheology in different 3D hydrogels and liver samples derived from a DEN-induced model for HCC). (**A**) Storage modulus of 3D hydrogels resembling a cirrhotic and fibrotic liver, grown with cells on day 1, 11 and 21. (**B**) Storage modulus of 3D hydrogels resembling a cirrhotic and fibrotic liver, seeded without cells, measured on day 1, 11 and 21. (**C**) Storage modulus of 3D hydrogels compared to the liver biopsies derived from a DEN-induced mouse model for HCC at day 1 and (**D**) day 21 of culture (n = 3, error bars = SD, ****p < 0.0001; ***p < 0.001, **p < 0.01, *p < 0.05).

To determine the stiffness of the biomimetic hydrogels used in our experiments, the storage modulus (kPa) was measured using parallel plate rheology of gels containing HCC-cells and of gels without cells (Fig. 1A,B). We obtained a significantly higher stiffness value for the cirrhotic 3D hydrogels compared to those corresponding to fibrotic gels, with the highest difference at the earlier time points (day 1 and day 11). As the experiment progressed, stiffness of the cirrhotic gel decreased, which was most pronounced in the condition without cells (Fig. 1B) and is thus likely due to gradual disposition of the gel^13^. However, even at day 21, cirrhotic gels containing cells obtained a significantly (p = 0.02) higher storage modulus fibrotic gels (Fig. 1A). To confirm whether these values were within a physiological range, rheology measurements were conducted on liver samples derived from healthy mice and from mice with DEN-induced liver cancer (Fig. 1C,D, Table 1). Healthy mouse livers had a stiffness of 2.8 kPa, while DEN-induced mouse livers with tumours had a stiffness of 4.4 kPa (Table 1). Overall, these data suggest that the storage modulus of the fibrotic and cirrhotic gels is within the same range as what is seen in vivo.

**Table 1:** Liver stiffness (kPa) determined via parallel plate rheology in 3D hydrogels and liver samples derived from a DEN-induced model for HCC.

Gel conditions (mouse condition)	Day 1	Day 11	Day 21	Mouse
Fibrotic (healthy)	3.017	1.919	2.796	2.862
Cirrhotic (DEN)	5.909	4.383	3.499	4.433

### Functional characterization

**Figure 2:**
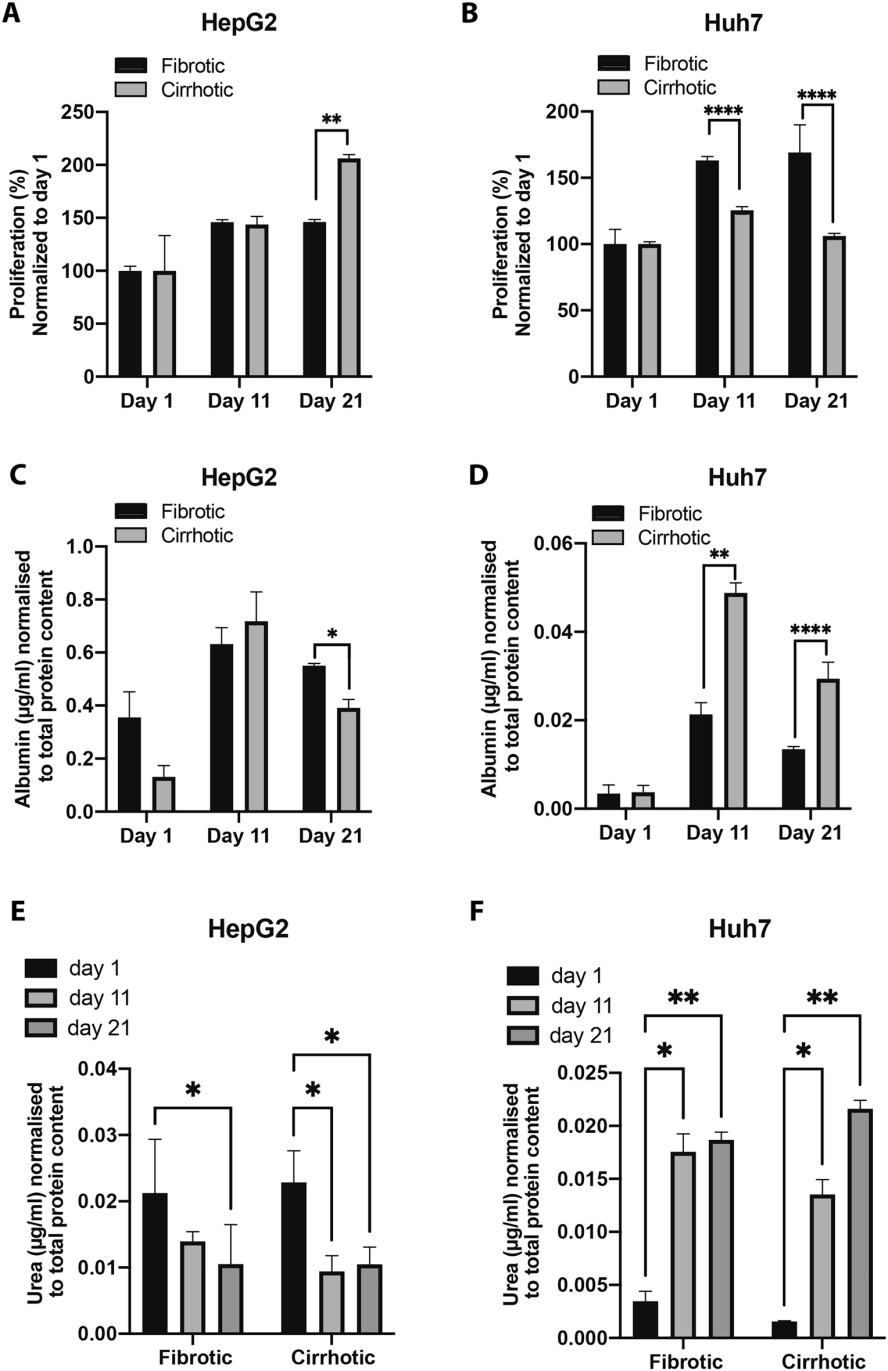
Viability, albumin, and urea content of cells grown in the 3D hydrogels resembling a fibrotic and cirrhotic liver. (**A**) Proliferation of HepG2 and (**B**) Huh7 cells. (**C**) Albumin normalised to total protein content of HepG2 and (**D**) Huh7 cells. (**E**) Urea normalised to total protein for HepG2 and (**F**) Huh7 cells. (n = 3, error bars = SD, ***p < 0.001, **p < 0.01, *p < 0.05).

It was essential that our biomimetic model not only recapitulates biophysical properties of the TME but also includes functional characteristics typically seen in a clinical setting. HepG2 and Huh7 cells embedded in hydrogels corresponding to a fibrotic and cirrhotic environment were evaluated on days 1, 11 and 21 in terms of viability, albumin synthesis and urea production. Both cell lines were viable in the 3D hydrogels and continued to proliferate throughout the duration of the study. The HepG2 line showed similar proliferation in both fibrotic and cirrhotic conditions on day 1 and 11. However, a statistically significant increase in proliferation was seen in the cirrhotic environment on day 21 in culture (Fig. 2A). Interestingly, Huh7-cells showed a significant reduction in proliferation at 11 and 21 days of culturing in the cirrhotic gels (Fig. 2B). HepG2 cells grown in a fibrotic and cirrhotic matrix, had similar albumin levels at days 1 and 11, but a significant decrease was noted at day 21 (Fig. 2C), which coincides with their increase in proliferation (Fig. 2A), similar as what is seen in previous studies^14^. Conversely, the Huh7 cell line showed a significant increase in albumin levels in the cirrhotic environment on days 11 and 21 compared to the fibrotic environment (Fig. 2D). In line with previous reports, albumin levels are highest at day 11 in all tested cell lines and matrix compositions, which then decreases after 21 days in culture^15^. Urea levels in the HepG2 cell line decrease over time in both fibrotic and cirrhotic conditions (Fig. 2E), whereas we monitored an increase over time in the Huh7 cell line in both conditions (Fig. 2F). No statistically significant differences were determined in urea concentrations between cells grown in a cirrhotic or fibrotic matrix, respectively.

### Spontaneous formation of spheroids and cells outside the hydrogels over time

**Figure 3:**
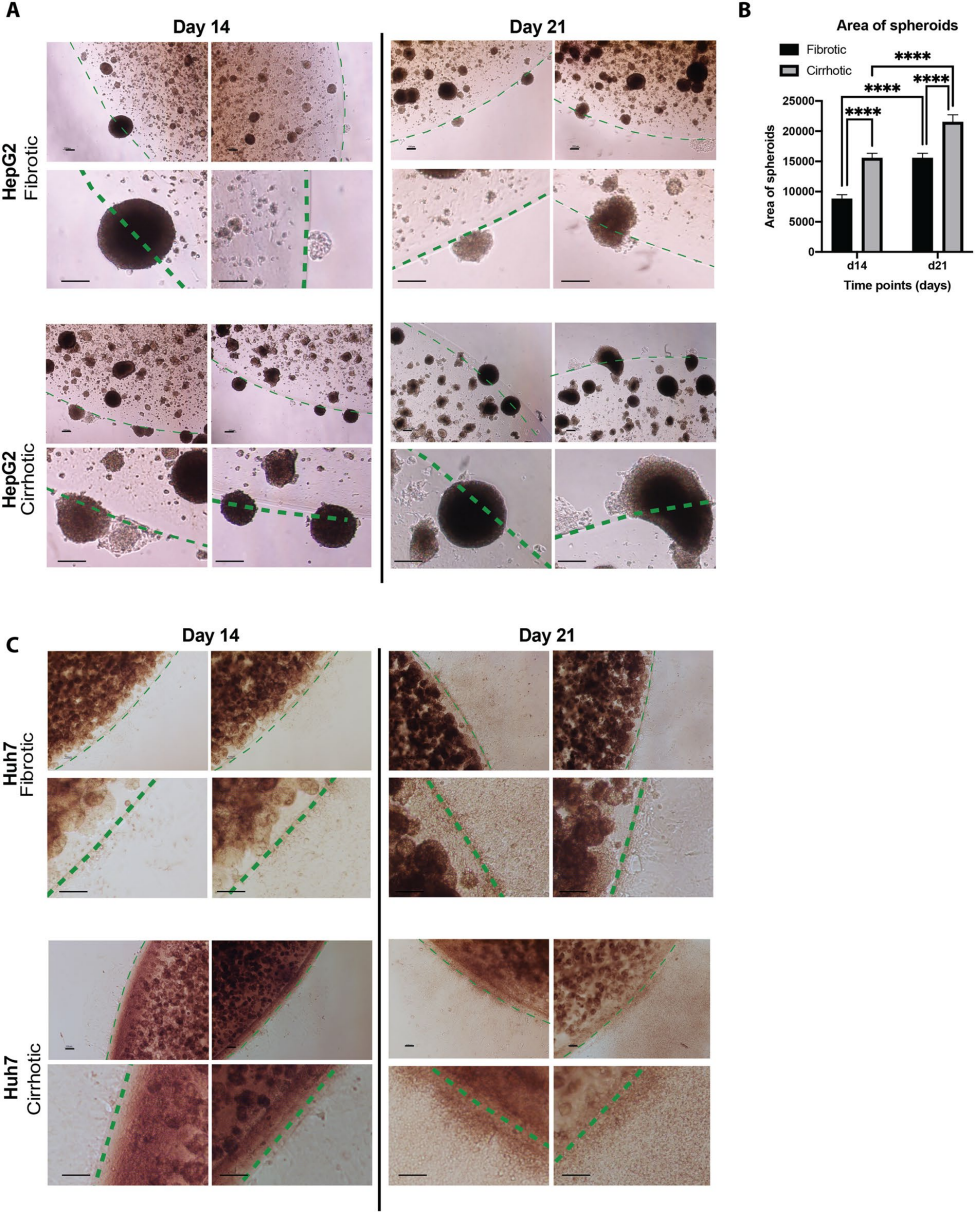
(**A**) Photomicrographs of tumour HepG2 spheroids forming in a 3D fibrotic and cirrhotic hydrogel after 14 and 21 days in culture. (**B**) Surface area of tumour nodules formed on day 14 and 21 in both fibrotic and cirrhotic environments. (**C**) Photomicrographs of Huh7 cells outside the hydrogel after 14 and 21 days in culture (dashed green line marks edge of gels, n = 150, error bars = SD, **p < 0.001, Scale bar = 100 µm).

On day 11 and 12 we noticed a spontaneous formation of tumour nodules outside the cirrhotic and fibrotic hydrogels seeded with HepG2-cells, respectively. Both size and number of these tumour nodules increased over time for both conditions (Fig. 3A,B). However, within the cirrhotic environment, these tumour nodules appeared slightly earlier (namely at day 11, versus day 12), and were also significantly larger in size when compared to the fibrotic environment (Fig. 3B). Upon evaluation of hydrogels containing Huh7-cells, we found that Huh7-cells migrated out of the hydrogels in both 3D conditions, yet migrating Huh7-cells did not form spheroids and remained growing as mono-layers (Fig. 3C).

### Composition of the ECM influences mRNA-expression of epithelial-to-mesenchymal transition and stemness markers and markers for endoplasmic reticulum stress

**Figure 4:**
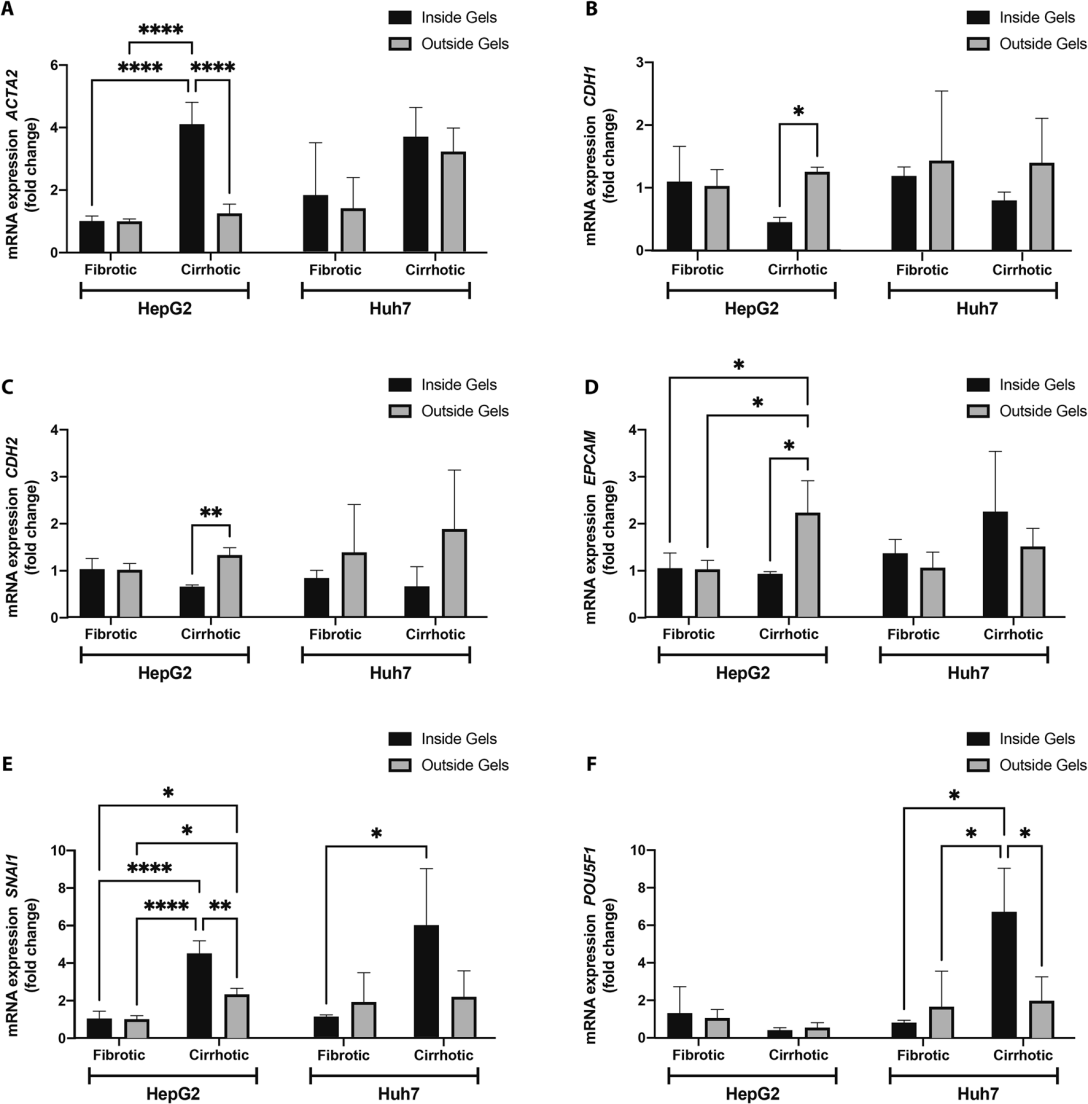
mRNA expression of EMT markers. (**A**) mRNA-expression of *ACTA2* (**B**) *CDH1* (**C**) *CDH2* (**D**) *EPCAM* (**E**) SNAI1 and (**F**) *POUF1* in HepG2 and Huh7 cells isolated from cells that appeared outside the gels and from cells that remained within the hydrogels (n = 3, error bars = SD, ****p < 0.0001, **p < 0.01, *p < 0.05).

Based on the formation of spheroids and occurrence of cells outside gels of HepG2 and Huh7 3D cultures, respectively, we set out to determine the effect of hydrogel composition on mRNA expression markers related to EMT, cancer stemness and endoplasmic reticulum (ER) stress. HepG2 and Huh7 cells inside and outside gels were collected from 3D fibrotic and cirrhotic conditions on day 25. HepG2-cells grown inside a hydrogel resembling a cirrhotic matrix were characterized by a significant increase in *ACTA2*-mRNA expression (Fig. 4A). Interestingly, when HepG2-cells migrated outside the cirrhotic hydrogels, they reduced *ACTA2*-mRNA expression. Although not statistically significant, HepG2 cells grown in cirrhotic gels were also characterized by a 46% reduction in *CDH1*-mRNA expression (Fig. 4B). These levels were restored in HepG2-cells that had escaped the cirrhotic hydrogels. *CDH2*-mRNA levels of HepG2-cells were also significantly lower in those grown within the hydrogels, compared to those outside the hydrogels (Fig. 4C) and *EPCAM*-mRNA levels were significantly increased in HepG2-cells that escaped the cirrhotic hydrogels (Fig. 4D). Overall, these results indicate a more epithelial phenotype in the cells found outside the hydrogels, compared to those inside the gels. No statistically significant changes were observed in *ACTA2, CDH1, CDH2 and EPCAM* mRNA expression in Huh7-cells grown between the tested experimental conditions. However, both HepG2 and Huh7 cells grown inside cirrhotic gels were characterized by a significant increase in mRNA-expression of *SNAI1* compared to those grown in fibrotic gels (Fig. 4E). Again, this was reduced in cells that grew outside the cirrhotic hydrogels (Fig. 4E). Similarly, mRNA-levels of *POU5F1* were increased in Huh7-cells grown inside cirrhotic gels, compared to those grown in fibrotic cells, with a significant decrease in those cells that escaped the hydrogel (Fig. 4F). *POU5F1-*mRNA-levels remained unaltered in HepG2 cells and were overall low.

As studies have shown that *POU5F1* (Oct4) is regulated by *EIF2AK3* (Perk)^16^ and we have previously shown that HepG2-cells and Huh7-cells have markedly different baseline and threshold levels of endoplasmic reticulum stress^17^, we measured mRNA-levels of *HSPA5, EIF2AK3* and *DDIT3 in* HepG2 (Supplementary Fig. 1a) and Huh7-cells (Supplementary Fig. 1b). Indeed, Huh7-cells grown in cirrhotic gels showed a marked increase of EIF2Ak3, which could have contributed to the increased mRNA-levels of *POU5F1*. This effect was not observed in HepG2-cells.

### The effect of ECM composition on drug response

**Figure 5:**
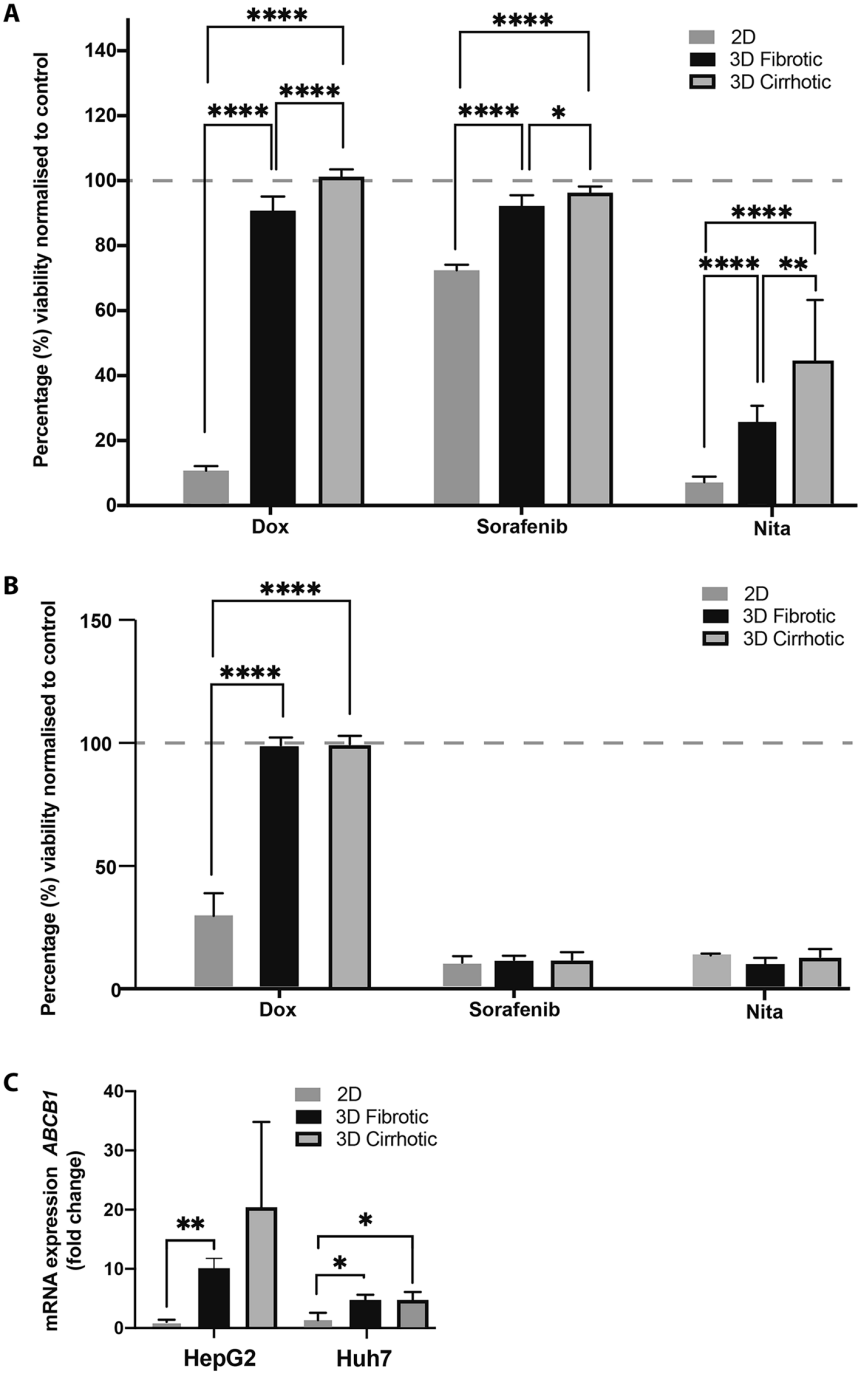
Effect of microenvironment on drug response to doxorubicin, sorafenib and nitazoxanide. (**A**) Percentage cell viability of HepG2 cells grown in 2D and 3D 96 h after treatment with Doxorubicin (1 µM), sorafenib or Nitazoxinide (10 µM). (**B**) Percentage cell viability of Huh7 cells grown in 2D and 3D 96 h after treatment with Doxorubicin (1 µM), sorafenib or Nitazoxinide (10 µM). (**C**) mRNA expression of ABCB1 in HepG2 and Huh7 cells grown in 2D and 3D. All values are normalised to the respective untreated control groups. (n = 3, error bars = SD, *p < 0.05, **p < 0.01, ***p < 0.001, ****p < 0.0001).

**Figure 6:**
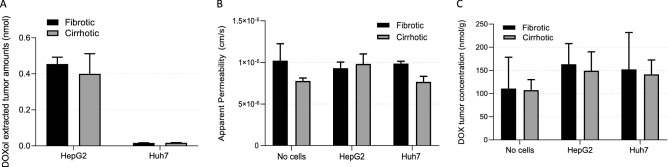
(**A**) Permeability of doxorubicin (1 mM) in fibrotic and cirrhotic gels without cells and with HepG2 or Huh7 cells. Doxorubicinol (**B**) and Doxorubicin (**C**) tumour concentration (nmol/g) in collected fibrotic and cirrhotic gels without cells or with HepG2 or Huh7 cells. (n = 3, error bars = SD.

To determine how 3D-culture conditions, as well as the fibrotic and cirrhotic environment influences drug response, HepG2 and Huh7 cells were cultured as 2D monolayers and in the 3D hydrogel models. Cells were exposed to the chemotherapeutic agent DOX (1 µM), the multi-kinase inhibitor Sorafenib and the oxidative phosphorilation-inhibitor Nitazoxanide (NITA) (10 µM) for 96 h. Following exposure, viability was measured using a resazurin reduction assay, and untreated control groups were analysed for *ABCB1* mRNA-expression. HepG2 and Huh7 cells grown in 2D, that were treated with DOX, sorafenib, NITA significantly decreased cell viability, as expected (Fig. 5A,B)^18,19^. HepG2-cells grown in 3D hydrogels were markedly less sensitive to DOX, sorafenib and NITA, when compared to cells grown in 2D. While DOX seemed to have very little or no cytotoxic effect on HepG2-cells grown in 3D, treatment with NITA led to over 60% reduction of HepG2-cells grown in 3D hydrogels (Fig. 6A). Interestingly, we observed a significant increase in the viability of the 3D cirrhotic group compared to the 3D fibrotic group of the HepG2 line when treated with DOX, suggesting growing the cells on a stiffer hydrogel matrix might have decreased their sensitivity to DOX. While Huh7-cells became more resistant to DOX-treatment when grown in hydrogels, this effect was interestingly not observed after Sorafenib and Nita-treatment, as all three conditions led to The datasets used and/or analysed during the current study available from the corresponding author on reasonable request. approximately 80% reduction in cell viability compared to untreated controls (Fig. 5B).

We then evaluated the expression of *ABCB1*, a gene that encodes for a P-glycoprotein that is part of the ATP-binding cassette (ABC) superfamily of transport proteins. Data from qPCR indicates a significant increase in the expression *ABCB1* of HepG2 cells grown in our 3D fibrotic environment compared to the 2D monolayers. A similar significant increase in *ABCB1* expression is seen when we compare Huh7 cells grown in, or 3D fibrotic and cirrhotic model compared to the 2D monolayers. Possibly, this could have contributed to the decreased response to DOX.

### Drug distribution and permeation study

To ensure that factors such as drug distribution and penetration are not responsible for the increased viability seen in our 3D model, we also evaluated the apparent permeability coefficients (P_app_) of DOX in our model both with and without cells at 21 days (Fig. 5). Doxorubicin permeability through the fibrotic gels was similar both with and without cells meanwhile the cirrhotic gels displayed slightly lower permeability for gels without cells or with Huh7 cells. The gels were collected after the experiment and the levels of DOX and its primary metabolite doxorubicinol were determined. Generally, higher levels of doxorubicin were found in the gels containing cells but no difference between fibrotic and cirrhotic gels were observed (Fig. 6C). Doxorubicinol was found in higher levels in HepG2 containing gels compared to Huh7 containing gels (Fig. 6B). The mass balance for all experiments was > 75% and no difference between gels or cells were observed.

## Discussion

Hepatic fibrosis is an integral part in the progression of chronic liver disease, ultimately leading to cirrhosis and hepatocellular carcinoma. This process is characterized by the activation of hepatic stellate cells and leads to the increased deposition of extracellular matrix (ECM) components, such as collagen^8^. The abundance of ECM will result in various physical and biomechanical changes in the microenvironment, including solid stress, increased matrix stiffness and interstitial hydraulic pressure^11^. Effectively mimicking altered biomechanical properties of disease states in vitro as well as in vivo has been challenging. The first goal of this study was to determine whether we could successfully apply an in vitro 3D hydrogel model to study the effect of matrix composition and liver stiffness on HCC. With slight modifications to our previously published model^7^, we embedded HepG2 and Huh7 cells into hydrogel formulations mimicking the liver stiffness of a fibrotic and cirrhotic environment during the development of HCC.

Changes in ECM composition are one of the characterizing features of the TME and it is widely accepted that this actively contributes to disease progression in different tumors^20,21^. This significance of altered ECM is seen in HCC patients, where increases in liver stiffness are associated with hepatic dysfunction, poor prognosis, and survival^22–24^. Recent studies have even shown that increased liver stiffness is a predictor for mortality and need for mechanical ventilation among patients hospitalized with COVID-19^25^. There is now a wealth of proteomic literature on ECM composition from both healthy and diseased liver showing that these ECM environments are a complex presentation of proteins. Work from Mazza et al. has shown that ECM harvested from decellularized healthy and cirrhotic livers can be used as an in-vitro cell culture platform for studying the effect of physiologically relevant liver ECM composition on cell behaviour^26^. During fibrosis, the ECM of the liver increases by eightfold, mainly due to the increased deposition of fibrillar collagens, fibronectin, elastin, laminins, and proteoglycans^25,27^. With fibrillary collagens being principal components of fibrotic scars, fibrinogen synthesis is also upregulated by two- to ten-fold^28^, which collectively results in a tenfold increase in liver stiffness during HCC development^6,11^. Results from the rheology data indicated that we successfully mimicked the liver stiffness seen during the onset of fibrosis and cirrhosis over 21 days, using physiologically relevant hydrogels consisting out of collagen I and fibrinogen. Not only were these results comparable to available literature but also to an in vivo DEN mouse model^17^. These results are significant as the development of in vitro tumour models requires recapitulating the complexity of the TME to improve predictive insights in tumorigeneses.

Alterations in matrix composition are not only associated with poor prognosis but also with hepatic dysfunction and increased proliferation in HCC^29^. It has also been shown in other tumours—such as pancreatic, breast and lung cancer—that increased matrix stiffness increases cancer cell proliferation^30–32^. We noticed a similar increase in proliferation of HepG2-cells grown in our model, when comparing cell viability and total protein content of cells grown in a cirrhotic environment to those grown in a fibrotic environment. However, we observed a decrease in proliferation at time point 11 and 21 in the Huh7-cells. Other studies have also observed this difference in proliferation rates of Huh7 and HepG2 cells grown on a stiffer matrix. The study of Schrader et al. noticed a 12-fold increase in proliferation of HepG2-cells grown on a stiff (12 kPa) matrix versus a soft (1 kPa) matrix, while Huh7 cells only exhibited a 2.7-fold increase. While HepG2 and Huh7 cells are both derived from well-differentiated tumours, several studies have pointed out their differences in terms of protein and gene expression profiles^18,33^, which is further confirmed in our study. Interestingly, one study has shown that different HCC-cell lines (including HepG2 and Huh7) have various levels of constitutive activity of beta-1-integrins and different regulatory mechanisms that mediate their response to collagens^34^. Therefore, the difference in fibrinogen-to-collagen ratio could have contributed to a different response in the two cell lines.

Albumin and urea levels are important markers of hepatocyte function in the liver. Studies have shown that culturing cells in 3D can increase albumin-mRNA expression, compared to those grown in 2D. Furthermore, it is known that the advanced stage of cirrhosis is characterized by impaired hepatocyte function, which leads do decreased albumin levels in patients and making albumin an important prognostic marker for cirrhotic patients^35^. A similar trend was seen in our HepG2 culture where albumin was significantly decreased in the cirrhotic environment compared to the fibrotic environment on day 21. Interestingly, this was not seen in the Huh7 cells, as a significant increase in albumin secretion was noted when Huh7-cells were grown in a cirrhotic matrix. Previous studies have also observed this difference in albumin secretion between HepG2 and Huh7 spheroids, suggesting that Huh7 spheroids synthesise low levels of albumin. While the Albumin Synthesis Rate of HepG2-cells peaks at 31.70 μg/10^6^ Nuclei/Day, Huh7 cells only have a 12.96 μg/10^6^ Nuclei/Day albumin synthesis rate under similar conditions^36^. Interestingly, Huh7-cells showed a time-dependent increase in urea production, while the opposite was observed in the HepG2-cells, which decreased urea secretion over time in the different matrices. This is in line with previous reports that also reported Huh7-cells to have up to a 42-times higher production of urea compared to HepG2-cells grown in similar conditions^37^. As we also observed differences in proliferation rate between HepG2 and Huh7 cells at the different time points, it is important to note that in vitro studies have shown that cellular proliferation can alter glutamine transport and metabolism in HCC-cell lines^38^. Moreover, baseline levels in carbamoyl phosphate synthase 1 (CPS1), the rate-limiting enzyme in the first step of the urea cycle, as well as hepatocyte nuclear factor-3-beta, a regulator of the CPS1-promotor, differ vastly between different HCC-cell lines^39^. Therefore, our results further warrant the importance of using multiple cell lines for HCC-research, in order to reflect the tumour heterogeneity found in HCC-patients.

HepG2 cells grown in the cirrhotic condition showed a higher population of tumour nodules forming outside of the cirrhotic gels over time when compared to those grown in the fibrotic environment. Similar higher populations of Huh7 cells were found outside of the cirrhotic condition over time, albeit without tumour nodule formation. The occurrence of cells or nodules outside the gels could reflect invasion or metastasis. One important contributor to invasion and metastasis, is EMT, which is driven—at least in part—by ECM components, soluble factors, and hypoxia^20,21,29^. To determine whether this was a contributing factor behind the population of cells found outside of the gels, we investigated known markers of EMT. The increase of *ACTA2 and SNAI1* in HepG2 cells grown in cirrhotic hydrogels, as well as the decrease of *CDH1* (the gene encoding for E-Cadherin) in cells grown in a cirrhotic matrix compared to those grown in fibrotic matrix, could indicate that the cirrhotic composition of the matrix induced a more pro-metastatic and mesenchymal phenotype. A similar effect is observed in Huh7-cells, where cirrhotic hydrogels induced expression of *SNAI1* and *POU5F1*, two important markers for cancer stemness. However, this mesenchymal or cancer stem cell line phenotype was not present in cells that migrated outside the gels. The switch towards a more epithelial phenotype of HepG2-cells which escaped cirrhotic hydrogels, compared to those growing inside the hydrogels is supported by increased *CDH2* (the gene encoding for E-Cadherin) and *EPCAM*-mRNA-levels in these cells. Similarly, Huh7-cells derived from the surrounding medium of gels showed a decreased expression of stem cell markers *SNAI1* and *POU5F1*, compared to those that remained inside the cirrhotic hydrogels. These results could indicate that while the cirrhotic hydrogels push the cells towards a mesenchymal phenotype, their epithelial phenotype is restored once they have migrated outside the gels. This is in line with the general hypothesis that EMT is necessary for the initial stages of metastasis, specifically for invasion and migration to distant tissues. However, the opposite phenomenon, mesenchymal-to-epithelial transition (MET), is needed for successful seeding and colonization at secondary sites^40^. Alternatively, it could be that the cirrhotic ECM provides active queues to push this mesenchymal and stem cell like phenotype, which is lost once the cells migrate outside the gels. Studies have shown that the ECM is a niche for normal and cancer stem cells, providing structural and biochemical support that drive cancer stemness and EMT. Collagen I has been shown to induce EMT through activation of NF-kB^41^, while fibrin(ogen) is known to bind to integrins which perceive the ECM biomechanical properties and transfer these signals to intracellular proteins such as Src tyrosine kinases^42^. More research is thus necessary to assess whether the cells lose their mesenchymal phenotype when they migrate outside the gels through the process of MET, or whether direct contact with the ECM is necessary to sustain this mesenchymal phenotype.

It is important to note the differences in expression of EMT markers between HepG2 and Huh7-cells under the different experimental conditions tested in this study. Although the exact molecular pathways remain to be elucidated, several studies have previously reported differences in the oncogenic profiles^18^ and stem cell markers between these two cell lines^43^. For instance, in our study, we show that the increase of *ACTA2* in cirrhotic hydrogels was more pronounced in HepG2-cells, which is in line with previous reports that have shown that Huh7-cells express low baseline levels of *ACTA2*^43^. In addition, HepG2-cells expressed very low levels of *POUF1,* while Huh7-cells exhibited a nearly sevenfold upregulation of *POUF1* when grown in cirrhotic hydrogels. Interestingly, previous studies have also observed different levels of *POUF1* between Huh7 and HepG2-cells and noted that HepG2-cells decrease *POUF1*-mRNA and Oct4-protein expression in response to FBS, which is not observed in Huh7-cells under these conditions^44^. In addition, we have previously shown that HepG2-cells and Huh7-cells have markedly different baseline and threshold levels of ER-stress^17^. Other studies have shown that ER-stress and specifically *EIF2AK3* can regulate Oct4-expression, thereby contributing to chemoresistance^16^. In our study, we found that Huh7-cells grown in cirrhotic gels showed a marked increase of *EIF2Ak3*, which could potentially have contributed to the increased mRNA-levels of Oct4. More research is necessary to confirm this interesting effect of ECM-composition on ER-stress and its potential link with EMT or cancer stemness.

Alterations in ECM composition may alter intracellular pathways leading to profound cellular reprogramming and thereby actively contribute to how the cells respond to chemotherapeutic agents^45^. At the same time, cancer stem cells are also known to show a high level of chemotherapeutic and radiation resistance. Specifically for HCC this could explain the high rates of recurrences and resistance to conventional chemotherapy^2^. Treatment with the chemotherapeutic agent DOX for 96 h showed nearly no effect on the cell lines grown in the different 3D-models, while the same dose elicited a nearly 90% reduction in cell viability when cells were grown in 2D. In line with these results, we found a significant increase mRNA-expression of *ABCB1* in our 3D model, compared to our 2D model for both cell lines. These results strengthen the notion that altered ECM stiffness is associated with increased resistance to DOX and poor survival and prognosis, but also that growing cells in 3D models efficiently recapitulates a clinically relevant drug resistance^46^. Interestingly, the response to sorafenib differed drastically between the two cell lines. The Huh7-cells were characterized by a much higher sensitivity to sorafenib in all experimental conditions compared to HepG2-cells. Previous studies have also noted that Huh7-cells are notably sensitive to sorafenib, and attributed this effect to an increased autophagic responsiveness^47^. Numerous studies have shown the strong connection between PERK-signalling pathways and autophagy^48,49^. Indeed, in our study, we also found that while Huh7-cells can strongly induce the expression of *EIF2Ak3* under cirrhotic conditions, this seems not the case for HepG2-cells. Possibly, this could have contributed to the different response of the two cell lines.

The results obtained from HepG2 and the Huh7 treated with NITA further demonstrate the influence of ECM-composition on drug response. Nitazoxanide is an FDA-approved broad-spectrum antiparasitic and antiviral that recently has shown promising results as a cancer treatment, specifically by targeting the hypoxic cells in different 3D models. Nitazoxanide exhibits the progression of solid tumours by altering various cellular process, namely drug detoxification, unfolded protein response, autophagy, and mitochondrial respiration^50^. This drug is most effective in hypoxic and nutrient deprived environments, for instance in the central core of solid tumours or in 3D tumour models^50^. In our study, we show improved efficacy of Nita compared to DOX in both cell lines, thus suggesting a promising alternative therapy for chemoresistant HCC-cells.

Firstly, this study shows that an in vitro 3D hydrogel model can be used to grow HCC-cells in a physiologically relevant matrix resembling a fibrotic and cirrhotic liver. Secondly, we successfully define how altering matrix stiffness affects tumour behaviour, as increased liver stiffness enhanced cancer cell proliferation, stimulated metastasis and induced a more chemoresistant tumour phenotype in HCC-cells. This could—at least in part—be explained by the activation of EMT and induction of cancer stemness when cells were grown on a stiffer, cirrhotic matrix. Finally, although cells grown in the 3D-models became nearly irresponsive to DOX-treatment, we propose an alternative therapy for chemoresistant HCC-cells, namely NITA, as this drug remained effective throughout all conditions.

## Materials and methods

### Cell culture

HepG2 (ATCC^®^ HB-8065™) and Huh-7 cells (gifted from Mårten Fryknäs, Uppsala University, Sweden) were routinely cultured at 37 °C with 5% CO_2_ in GlutaMAX™ supplemented, high glucose Dulbecco modified eagle medium (DMEM) (31966047, ThermoFisher Scientific, Stockholm, Sweden) with 1% antibiotic antimycotic solution (A5955-100ML, Sigma-Aldrich, Darmstadt, Germany) and 10% fetal bovine serum (FBS) (10270106, ThermoFisher Scientific, Stockholm, Sweden). No FBS was used during starvation. Misidentification of cell lines were checked at the Register of Misidentified Cell Lines and chosen cell lines were not on the list. Extracted DNA from all cell lines is sent yearly to Eurofins Genomics (Ebersberg, Germany) for cell line authentication using DNA/STR-profiles. Authentication confirmed the correct identity of our cell lines and tested negative for mycoplasma contamination.

For 2D cell culture, a HepG2 and Huh7 single cell suspension was prepared by trypsinization and seeded into 96-well plates at a seeding density of 1.5 × 104 cells/ml, with each well containing 3000 cells in 200 µl culture medium. For 3D cell culture* a* modified setup of cells embedded into a hydrogel consisting of fibrinogen (From bovine plasma, F8630-5G, Sigma-Aldrich, Darmstadt, Germany) and collagen (Rat-tail type I, 50201, Ibidi, Lund, Sweden) were prepared as previously described^7^ (Table 2).

**Table 2:** Formulation composition, correspondence to stage of liver disease and known liver stiffness values and measured stiffness values^7^.

Formulation concentration (mg/mL)		Stage of liver disease	Liver stiffness values (kPa)	
Fibrinogen	Collagen		Literature	Rheology data for model day 1
10	2	Fibrosis	≥ 2–4	3
30	2	Cirrhosis		6

Briefly, a stock solution of fibrinogen (60 mg/ml) was prepared. The HepG2 or Huh7 cells were prepared as single cell suspensions, counted, and diluted to 2 × 10^6^ cells/ml. The diluted cell suspension was centrifuged at 300*×g* for 3 min, and the supernatant removed. Hydrogels were added to the cells and 200 µl of the gel was seeded onto 12-well plates. Crosslinking of the gels was performed in the culture hood for 15 min at room temperature, followed by 45 min at 37 °C within a CO_2_ incubator. Growth medium was subsequently added to each well and replenished every second day. Gels were maintained for 21 days prior to experimentation, unless stated otherwise.

### Animal study

Five-week-old male sv129-mice were injected every other week with 35 mg/kg diethyl nitrosamine (DEN) or equal volumes of saline. In this model, tumours occur after 25 weeks, and mice livers were sampled post-mortem after 28 weeks^17^. All methods were approved by the Uppsala Ethical Committee for Animal Experimentation (DNR 5.8.18-0089/2020) and all experiments were performed in accordance with relevant guidelines and regulations. Reporting in the manuscript follows the recommendations in the ARRIVE guidelines.

### Rheology

All measurements were done in triplicate. Hydrogels were measured using a Discovery Hybrid Rheometer 2 (TA instruments, Sollentuna, Sweden. All measurements were performed as frequency sweeps from 0.02 to 2 Hz and 0.267% shear strain to study the differences in storage modulus as an indication of stiffness (data compared at 1.1 Hz frequency). The hydrogels were measured on day 1 of preparation, 11 and 21, both with and without cells to determine any potential cell mediated remodelling. For mouse liver samples, livers were collected from the left liver lobe using an 8 mm and biopsy punch. Samples were submerged in PBS and kept on ice, until rheology measurements. All measurements were performed at 37 °C, with a constant axial force of 0.1 N using an 8 mm diameter parallel plate stainless steel geometry.

### Resazurin reduction assay

Viability and proliferation were measured using a Resazurin reduction assay, following manufacturer’s guidelines. For 2D cultures culture medium was removed and cells were washed twice with 200 µl PBS. Starvation medium was added, and cells were incubated for 2-h. Following incubation, starvation medium was removed and 200 µl of the Resazurin solution was added to the cells and incubated overnight. For 3D cultures, culture medium from the gels were removed, and the cells washed twice with PBS and 1 ml starvation medium was added. After a 2-h incubation at 37 °C, starvation medium was removed and 1 ml Resazurin solution was added to each well, followed by overnight incubation at 37 °C. Following incubation 200 µl was transferred from each well into a Corning black clear bottom 96-well plate (Sigma-Aldrich, Darmstadt, Germany). Fluorescence was read using a FLUOstar Omega microplate reader (BMG Labtech) at excitation and emission wavelengths of 485 and 550 nm, respectively.

### Collagenase-dispase cell isolation

To isolate cells from hydrogel formulations, cell culture medium was removed, and the gels washed twice with 1 ml PBS. A volume of 700 µl collagenase-dispase (1 mg/ml) in PBS, was added to each gel, and the gels were incubated at 37 °C for 90 min. To assist with the dissociation step, gels were manually disrupted with a pipette at 20 min intervals. Once the gel was completely digested, the reaction was inactivated by adding 30 µl EDTA (10 mM). The resulting cell suspension was collected and centrifuged for 15 min at 1000×*g*. Following centrifugation, supernatant was removed, and cell pellet was resuspended in lysis buffer or culture medium depending on the downstream application.

### Protein content

Protein content was determined using the Pierce™ BCA Protein Assay kit (23225, ThermoFisher, Stockholm, Sweden), according to manufacturer's recommendations. Cells from hydrogels were isolated using collagenase-dispase and transferred to 2 ml collection tubes. Cell suspensions were subsequently centrifuged for 5 min at 500×*g*. Culture medium was aspirated, and cells were washed twice in PBS followed by lysis in 300 µl RIPA buffer supplemented with protease inhibitors for 20 min on ice. Samples were then centrifugated at maximum speed for 10 min and the supernatant was collected and transferred to a clear flat bottom 96-well plate.

### Albumin enzyme-linked immune sorbent assay (ELISA) to determine functionality

Cell culture medium from 3D hydrogel cultures were collected on day 1, 11 and 21. Samples were centrifuged for 2 min at 5000×*g* and the supernatant was transferred to a new collection tube and frozen. Albumin levels were measured by means of the Human Albumin (ALB) ELISA kit (EHALB, ThermoFisher, Stockholm, Sweden), following manufacturer´s guidelines with a 1:500 dilution of sample. Urea levels were measured by means of the Urea Nitrogen (BUN) colorimetric detection kit (EIABUN, ThermoFisher, Stockholm, Sweden), following manufacturer’s guidelines but without sample dilution. The averages from three biological replicates were used for calculations. All data was normalised to total protein content.

### Drug response and experimental group setup

The response to DOX (1 µM) and NITA (10 µM) was determined in the 2D-setup and hydrogel formulations. HepG2 and Huh7 cells were seeded onto black flat clear bottom plates and left overnight to attach. Three-dimensional hydrogel formulations were prepared and maintained for 21-days. Prior to drug treatment, culture medium was removed and both 2D and 3D cultures were washed twice with PBS. Starvation medium was added for 2 h, and cultures were subsequently incubated at 37 °C for 2 h. Following incubation, starvation medium was removed, and cultures were treated with DOX and NITA in low glucose medium (3 mmol/l) and pH 6 for 96 h. All measurements were done in triplicate.

### Drug distribution and permeation study

The permeability of DOX through the 3D cell model was assessed using an experiment inspired by an established protocol^51^. Cell culture media buffered with 25 mM HEPES (pH = 7.4) was used to maintain both donor and acceptor side pH. DOX (1 mM) was added to the donor side (insert) of the model. Samples (100 µl) were taken every 30 min from the receiver side (well) and an equivalent volume of fresh cell media was added (i.e., sample and replace method). The 12-well plates were gently agitated (100 rpm) and kept at 37 °C throughout the experiment, except for when sampling at room temperature. After 2 h the permeability experiment was ended, the donor side sampled (50 µl), the hydrogel washed with PBS and collected using a spatula and the receiver media pH measured. DOX and its main metabolite doxorubicinol (DOXol) were quantified using a previously published UPLC-MS method^52^. Briefly, the analytes of interest were extracted overnight from receiver side samples, as well as from the collected hydrogels, using protein precipitation with ice-cold acetonitrile containing set levels of isotopically labelled internal standards (1 µM). Donor side samples were diluted by a ten-fold in cell culture media before being treated similarly to receiver side samples. Data were processed in TargetLynx (MassLynx V4.1, Waters Corporation, Milford, MA, USA) using linear curve fitting (weighting factor of 1/x) of the peak area ratio (analyte:internal standard) as a function of the analyte concentration. Linear calibration curves (R^2^ > 0.99) in cell culture media for both DOX and DOXol were constructed between 0.125 and 25 µM and the lowest point in the calibration curve (125 nM) corresponded to the lowest limit of quantification (LLOQ). Apparent permeability coefficients (P_app_, cm/s) were calculated according to previously described equations^53^. Mass balance (%) was calculated based on donor and receiver amounts. DOX and DOXol hydrogel concentrations (nmol/g) were calculated by dividing the quantified extracted amount (nmol) from the gel by the weight of the collected gel (g).

### Plannimetry

Photomicrographs were taken of spheroid formation in hydrogels containing HepG2 cultures on day 14 and 21 using an Olympus IX81 motorized microscope and an Olympus DP71 camera. A total of 150 spheroids were measured in each condition at both time points using Fiji Image J software.

### Quantitative RT-PCR

Cells were either isolated from the gels using the collagenase-dispase isolation method described above, or by centrifugation of the cell culture medium which contained cells or spheroids that migrated outside the gels. RNA was then isolated using the EZNA^®^ RNA isolation Kit II (R6934-02, VWR, Spånga, Sweden). RNA-concentration and purity were evaluated using Nanodrop, followed by reverse transcription of 300 ng mRNA using the iScript select cDNA synthesis kit (1708897, Bio-rad, Solna, Sweden). Amplifications were done using primers summarized in Table 3. In all instances, mRNA-expression was normalized to housekeeping gene GAPDH, using the delta-delta-CT method to calculate fold change of three biological replicates.

**Table 3:** Overview of primers used in this study.

Protein	Forward	Reverse	Function
SNAI1	TCTAGGCCCTGGCTGCTAC	TGACATCTGAGTGGGTCTGG	EMT
CHD2	CTCCAGGGGACCTTTTCCT	CCGAGATGGGGTTGATAATG	EMT
CDH1	GAACGCATTGCCACATACAC	ATTCGGGCTTGTTGTCATTC	EMT
ACTA2	GACAGCTACGTGGGTGACGAA	TTTTCCATGTCGTCCCAGTTG	EMT
EPCAM	TGCAGTCCGCAAACTTTTACTA	ATAACCTGCTCTGAGCGAGTG	EMT, cancer marker
POU5F1	CCTCGCTTTCCCTAGCTCTG	TAGTCGCTGCTTGATCGCTT	Stem cell marker
ABCB1	AGAGCGGAGGACAAGAAGGTG	TGTTCTGGCTTCCGTTGCAC	Drug resistance

### Statistics and data availability

Unpaired, two-tailed Student’s T-test or one-way analysis of variance (ANOVA) followed by Tukey’s multiple comparison test was performed using GraphPad Prism version 8.0 to determine statistical significance. P-values < 0.05 were considered statistically significant. Experiments were done in at least three biological replicates, which we define as parallel measurements of biologically distinct samples taken from independent experiments. Technical replicates we define as loading the same sample multiple times on the final assay. Outliers were kept in the analyses, unless they were suspected to occur due to technical errors, in which case the experiment was repeated. The datasets used and/or analysed during the current study are available from the corresponding author on reasonable request.

## Supplementary Information

Supplementary Figure 1.

Supplementary Legends.
