# Supplementary figures and images for: Influence of extracellular matrix composition on tumour cell behaviour in a biomimetic in vitro model for hepatocellular carcinoma
Source: Sci Rep. 2023 Jan 13;13:748. doi: 10.1038/s41598-023-27997-3 (PMC9839216; doi:10.1038/s41598-023-27997-3)

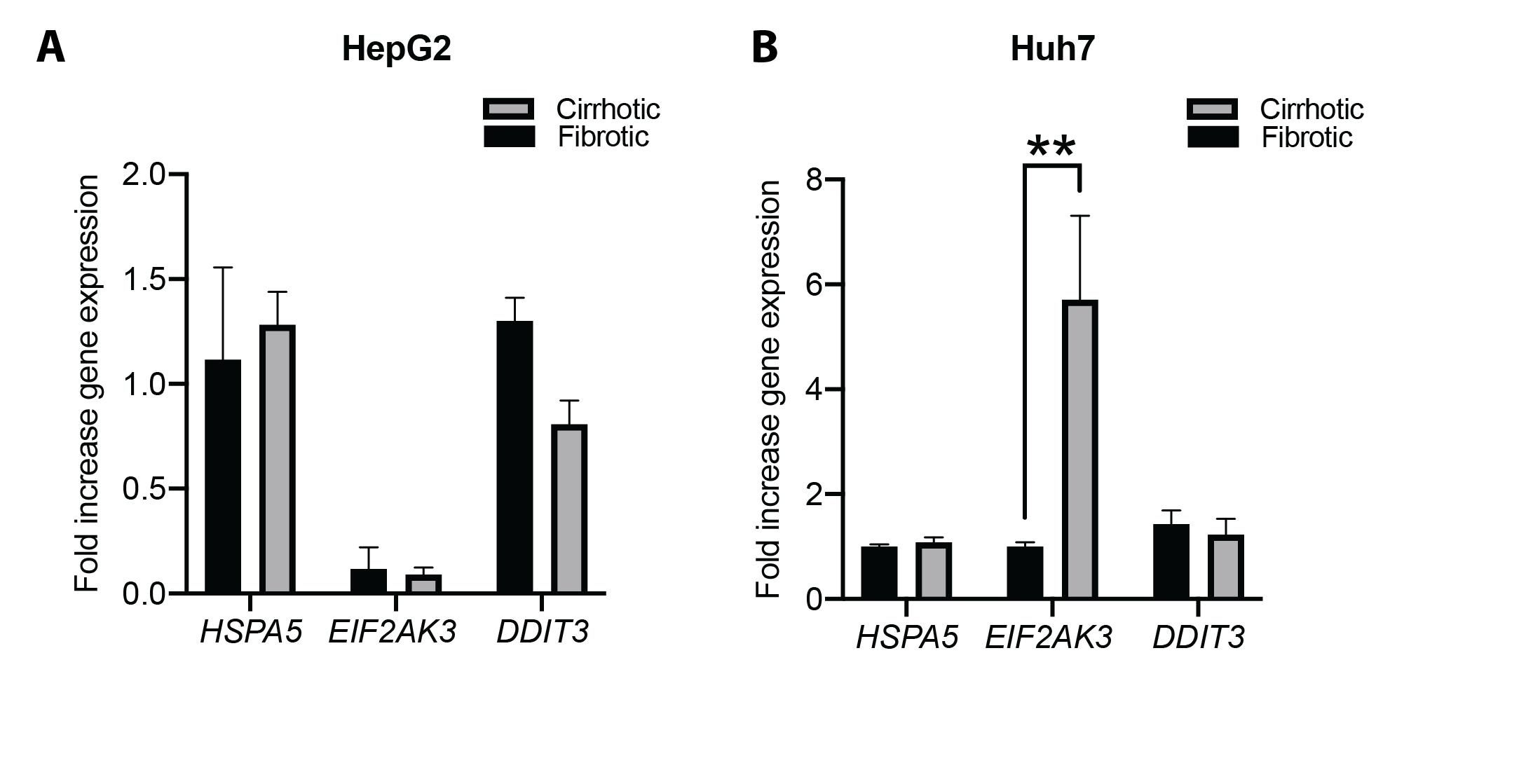

Supplement: Supplementary file 1 — Supplementary Figure 1. [file 41598_2023_27997_MOESM1_ESM.png]
