# Supplementary material for: Influence of extracellular matrix composition on tumour cell behaviour in a biomimetic in vitro model for hepatocellular carcinoma
Source: Sci Rep. 2023 Jan 13;13:748. doi: 10.1038/s41598-023-27997-3 (PMC9839216; doi:10.1038/s41598-023-27997-3)
Supplement: Supplementary file 2 — Supplementary Legends. [file 41598_2023_27997_MOESM2_ESM.docx]

*Supplementary figure 1: mRNA expression levels of ER-stress related genes HSPA5, EIF2AK3 and DDIT3 in (A) HepG2 and (B) Huh7 cells.* (n=3, error bars=SD, ** = p < 0,01)
